# Simultaneous Determination of Deoxynivalenol, Its Modified Forms, Nivalenol and Fusarenone-X in Feedstuffs by the Liquid Chromatography–Tandem Mass Spectrometry Method

**DOI:** 10.3390/toxins12060362

**Published:** 2020-06-01

**Authors:** Łukasz Panasiuk, Piotr Jedziniak, Katarzyna Pietruszka, Andrzej Posyniak

**Affiliations:** Department of Pharmacology and Toxicology, National Veterinary Research Institute, Al. Partyzantów 57, 24-100 Puławy, Poland; piotr.jedziniak@piwet.pulawy.pl (P.J.); katarzyna.pietruszka@piwet.pulawy.pl (K.P.); aposyn@piwet.pulawy.pl (A.P.)

**Keywords:** type B trichothecenes, modified mycotoxins, isomer separation, method validation

## Abstract

A liquid chromatography-tandem mass spectrometry method was developed for simultaneous determination of deoxynivalenol (DON), 3-acetyldeoxynivalenol (3Ac-DON), 15-acetyldeoxynivalenol (15Ac-DON), DON-3-glucoside (DON-3Glc) nivalenol and fusarenone-X in feedstuffs. Different techniques of sample preparation were tested: solid-liquid-extraction, QuEChERS, solid phase extraction with OASIS HLB columns or immunoaffinity columns and a Mycosep 225 Trich column. None of the six immunoaffinity columns tested showed cross-reactivity to all of the mycotoxins. Surprisingly, the results show that if the immunoaffinity columns bound 3Ac-DON, then they did not bind 15Ac-DON. The most efficient sample preparation was achieved with a Mycosep 225 Trich column clean-up. The chromatography was optimised to obtain full separation of all analytes (including 3Ac-DON and 15Ac-DON isomeric form). The validation results show the relative standard deviations for repeatability and reproducibility varied from 4% to 24%. The apparent recovery ranged between 92% and 97%, and the limit of quantification described a 1.30 to 50 µg/kg range. The method trueness was satisfactory, as assessed by a proficiency test and analysis of reference material. A total of 99 feed samples were analysed by the developed method, revealing the presence of DON and DON-3Glc in 85% and 86% of examined animal feeds, respectively at concentrations between 1.70 and 1709 µg/kg. The ratios DON-3Glc to DON in the surveyed feedstuffs were from a low of 3% to high of 59%.

## 1. Introduction

Type B trichothecenes are a group of mycotoxins produced by *Fusarium* genera (*F. graminearum* and *F. culmorum*) and are currently some of the most prevalent and important contaminants of cereals in the field. So far, the best-known toxins in feedstuffs in this group are deoxynivalenol (DON), nivalenol (NIV) and fusarenone-X (FUS-X) [1]. These toxins are resistant to milling, processing and heat and, therefore, it is very hard to eliminate them from the feed chain [2]. Among type B trichothecenes, DON is the most prevalent and hazardous mycotoxin and its occurrence can cause many adverse health effects in animals, such as feed refusal, emesis, suboptimal weight gain and diarrhoea, which can lead to economic losses. All animal species evaluated to date are susceptible to DON in this order of vulnerability: pigs > mice > rats > poultry ≈ ruminants [3]. 

Moreover, in recent years, the occurrence of so-called “modified mycotoxins” is an increasing concern in feed and food safety, since they remain undetected in testing for their parent mycotoxin [4]. Modified forms of DON can be formed by fungi as the acetylated derivatives: 3-acetyldeoxynivalenol (3Ac-DON) and 15-acetyldeoxynivalenol (15Ac-DON) or as a form of the parent toxins conjugated with glucose (DON-3glucoside; DON-3Glc). DON-3Glc is produced as part of the defense system of a plant infected by toxigenic fungi. These toxic compounds can transform into their parent toxin by hydrolysis in the mammalian digestive system [5]. DON-3Glc has lower toxicity than its precursor, while both acetylated forms possess equivalent or much stronger toxicity to animals, and their conversion into their native form also cannot be excluded [6]. Moreover, recently published papers show that 15Ac-DON has a higher toxicity then 3Ac-DON [7]. 

At the time of writing, the guidance values for the native form of DON in feedstuffs are set down in Commission Recommendation 2006/576/EC with amending Recommendation 2016/1319 [8,9], but other toxins (DON-3Glc, 3Ac-DON, 15Ac-DON, NIV and FUS-X) are not included. Consequently, the occurrence of DON modified forms would imply an underestimation of the level of DON contamination in feedstuffs. Nevertheless, in recent guidelines the European Food Safety Authority (EFSA) has launched calls for data on occurrence in food and feed of DON, NIV and modified DON mycotoxins to enable drafting of a scientific opinion on mycotoxins with respect to food and feed safety [10,11,12].

The important issue is the simultaneous determination of DON and its modified forms. Various methods for their analysis in cereals and feedstuffs have been reported, such as liquid chromatography (LC) coupled with tandem mass spectrometry (MS/MS) [13,14,15,16], fluorescence detection (FLD) [17], photodiode-array detection (PDA) [18,19,20], and ultra-high-performance supercritical fluid chromatography-tandem mass spectrometry (UHPSFC-MS/MS) [21]. Moreover, in recent years the introduction of high-resolution mass spectrometers (HRMS) has allowed screening of non-target compounds, novel compound identification and retrospective data analysis [22]. In previously published studies for chromatographic separation, authors used mostly C18 columns [13,16,19,23].

Different sample preparation techniques and clean-up approaches have been used in feedstuffs: solid-liquid-extraction (SLE) without clean-up [16,23], the quick, easy, cheap, effective, rugged and safe (QuEChERS) technique [24], solid-phase-extraction (SPE) [14] and immunoaffinity columns (IAC) [17,25,26]. While SLE is frequently applied for multi-mycotoxin analysis in feedstuffs, the inclusion of clean-up strategies in the procedure could increase the sensitivity of the method, as well as decrease high matrix effect for the difficult matrix. 

One of the challenges in this analysis is the chromatographic separation of the 3Ac-DON and 15Ac-DON isomers, which only differ in structure by the position of the acetyl group. They have the same daughter ions, so it is crucial to fully separate them by LC-MS/MS for accurate quantification. Therefore it is important to derive validated methods for accurate assessment of exposure to DON and its metabolites by determining their levels in feedstuffs (due to their different toxicities). 

The paper describes the development of a method for determination of DON, its modified forms, NIV and FUS-X with particular reference to the following aspects: full separation of all analytes (including isomeric forms of acetylated DON), comparison of different strategies for sample preparation and clean-up (SLE, QuEChERS, SPE with OASIS HLB columns or IACs and a Mycosep 225 Trich column) as well as verification of the method in a proficiency test (PT) and with naturally contaminated feed samples.

## 2. Results and Discussion

### 2.1. LC-MS/MS Optimisation

The optimisation of LC-MS/MS parameters was accomplished by directly applying tuning solutions of the selected mycotoxins at concentrations of 1 µg/mL each, using 0.1% CH_3_COOH and MeOH as a mobile phase. The MRM mode was used and the analytes were ionized in both positive (ESI^+)^ and negative modes (ESI^−^) (Table S1). In the case of positional isomers, proper quantification of 3Ac-DON and 15Ac-DON cannot be achieved through the difference of the product ion in MRM mode. In positive mode (339 *m*/*z*) the most abundant parent ion was the same for both analytes, but different product ions were chosen (339/231.2 and 339/279.1 for 3Ac-DON and 339/321.2 and 339/261.2 for 15Ac-DON) [27,28]. However, other studies show higher ionisation in positive mode with NH4^+^ adducts for 3Ac-DON and 15-AcDON [13,23] or in negative modes [14,16]. If two different daughter ions for both isomeric forms are be chosen, positive false results could sometimes occur. For proper qualification and quantification it is therefore essential to have successful baseline separation of analytes.

### 2.2. Chromatographic Separation

**Figure 1:**
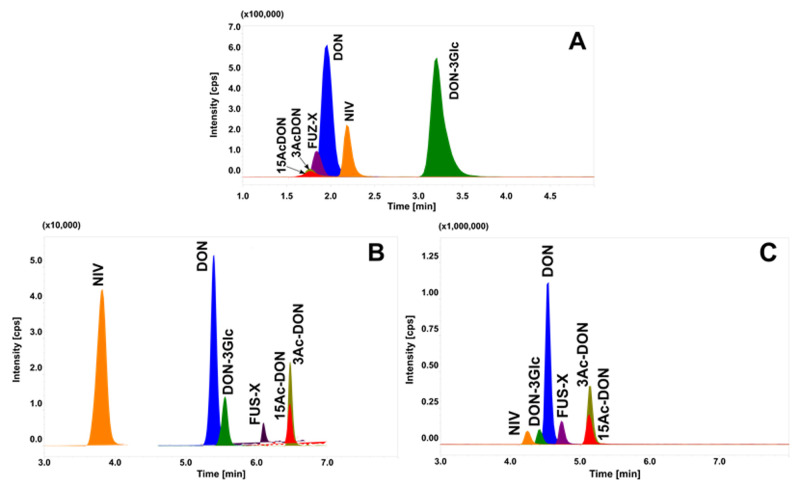
LC–MS/MS chromatograms of analysed toxins, tested in the same mobile phase but at different chromatographic columns: (**A**) Phenomenex Luna HILIC (**B**) Phenomenex Kinetex Biphenyl 100 × 2.1mm, 1.7 µm; (**C**) Phenomenex Kinetex C18 100 × 2.6 mm, 2.1 µm.

**Figure 2:**
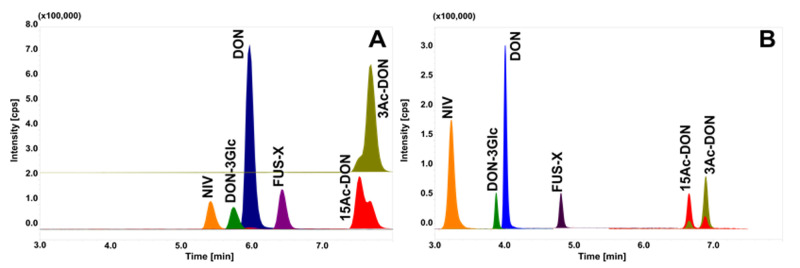
Chromatographic separation of tested compounds on the same column (Phenomenex Luna ^®^ Omega C18 100 × 2.1, 1.6 µm) with different organic mobile phase: (**A**) MeOH; (**B**) ACN.

Due to the different polarities of the analysed toxins, several chromatographic columns (Table S2) and mobile phases were tested. Based on the literature data, hydrophilic interaction liquid chromatography (HILIC) was tested as a first choice. Satisfactory separation on DON and DON-3Glc was achieved with Luna HILIC column (Phenomenex, Torrance, CA, USA), For other toxins no satisfactory retention times were obtained, and poor separation of all analytes was observed (Figure 1A). Kinetex C18 and Kinetex Biphenyl columns (Phenomenex) were also compared (Figure 1B,C). Although, these columns enable DON, DON-3Glc, NIV and FUS-X to be separated, 3Ac- and 15Ac-DON were not. In our study, the best results were achieved using a Luna Omega Polar C18 column (Phenomenex; 100 × 2.1; 1.6 µm) which is designed for polar compounds. Application of this column with MeOH as the organic mobile phase allowed all analytes to be separated except 3Ac and 15Ac-DON, their peaks still broadening (Figure 2A), and co-eluted. 

To obtain full separation of peaks, ACN was used as an organic mobile phase with specific gradient mode (2–6 min with an almost isocratic gradient from 15–18% of ACN) (Figure 2B). Moreover, the choice of ACN shortened retention time for all analytes, as well as the time of analysis. To date a lot of studies have described the chromatographic separation of DON, its modified mycotoxins, and other type B Trichothecenes in different biological matrixes [13,14,16,18,27,28,29,30,31,32,33,34]. Nevertheless, most of the authors did not achieve baseline separation of acetylated forms of DON. Only a few studies described the full chromatographic separation of these compounds [17,35,36]. Yoshinari et al. (2013) obtained retention times for 3Ac-DON and 15Ac-DON of 5.48 and 5.60 min, respectively. In a different study Goncalves et al. [17] achieved partial separation of the isomeric form (both peaks co-eluted). Contrary results were demonstrated by Slododchikova et al. [37] who concluded that the best for separation of the isomeric forms of acetylated DON was a pentafluorophenyl column and MeOH as the mobile phase.

In conclusion, the usage of a Phenomenex Luna Omega Polar C18 column (100 × 2.1; 1.6 µm) column with the combination of 0.2% CH_3_COOH with ACN as the mobile phase in a specific gradient mode achieves full separation of all tested compounds with a total run time of 12 min (Figure 2B).

### 2.3. Sample Preparation

#### 2.3.1. IAC Testing

**Figure 3:**
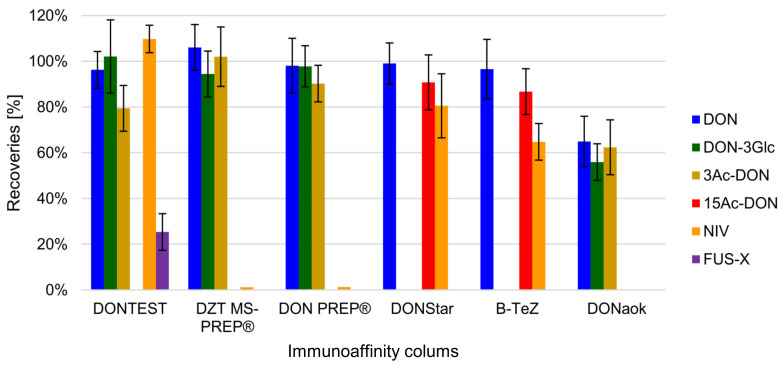
Mycotoxins recoveries obtained with IACs columns available on the market, obtained from different suppliers.

Six commercially available IACs were tested (DONTest, DZT MS-PREP, DON PREP, B-TeZ IAC, DONStar, and DONaok). Cross reactivity with the modified mycotoxins depended on the immobilized antibody [38]. While all the IACs showed excellent recovery for DON (Figure 3), none of them bound all DON metabolites and other toxins. For DON-3Glc, DONTEST, DZT MS-PREP, DON PREP and DONaok cross-reacted which is in line with other researchers’ results [17,20,39]. Contrary to findings in other papers, none of IACs tested retained 3Ac-DON and 15Ac-DON simultaneously. Our results showed that if the antibodies bound 3Ac-DON, they did not bind 15Ac-DON. These results are in disagreement with those of another study, where a DONTEST column was used to determine DON derivatives with good recoveries (over 80%), although with HPLC post-column derivatisation and fluorescence detection [17]. In turn, Versilovskis et al. [39] demonstrated that DZT MS-PREP, DONPREP and DONaok cross-reacted with 15Ac-DON, although the recoveries obtained were low, and did not exceed 25%. However, the chromatographic method did not separate 3Ac- and 15Ac-DON and the LC-MS/MS method was based on the MRM of the [M^+^H^+^] ions for both isomers, which can lead to false positive results. Lack of fully separated analytes could be a reason why other authors achieved discrepancy results. Moreover, we also checked possible cross-reactivity IACs with others B-trichothecene: NIV and FUS-X. From all tested IACs DONTEST, DONSTAR and B-TEZ bound NIV. Our results are in agreement with Uhlig et al. [40] where the authors highlighted that DONTEST retained NIV. For FUS-X, only DONTEST IAC bound toxins (25%), which was not tested in any previous papers. Because the DONTEST IAC showed best results of all the tested columns (but not full satisfactory), it was chosen for further evaluation.

#### 2.3.2. Comparison of Different Strategies for Sample Preparation and Clean-Up

**Figure 4:**
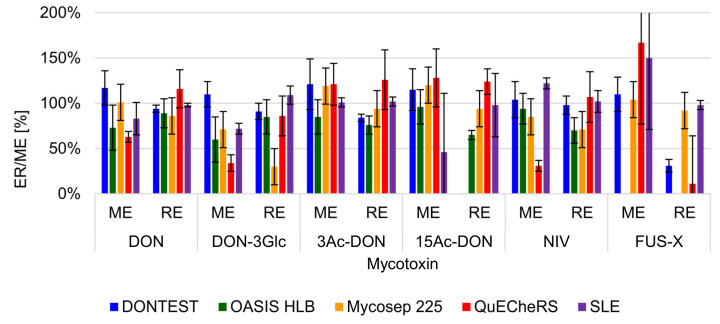
Extraction recovery (ER) and matrix effect (ME) of the tested methods for sample preparation.

The suitability for extraction and clean-up of DONTEST IAC, OASIS HLB and Mycosep 225 was tested by using them according to the manufacturer’s instruction. The QuEChERS technique and SLE were prepared based on our previous experience [41,42]. As is shown in Figure 4 the best results were obtained for Mycosep 225 columns which are dedicated products for Trichothecene analysis. In our study, obtained ER were in the range of 86–94%, except for DON-3Glc where 30% recovery was achieved. However, application of matrix-matched calibration curves could effectively compensate for recovery losses (see the Method Validation section) [43]. Lower recovery of DON-3Glc using a Mycosep 225 column was previously reported [30]. 

A significant advantage of these columns was the lowest ME for all compounds (71–120%). “Push-through” columns were previously used with grain extracts [30], where the authors reported recovery for NIV, DON and FUS-X in the range of 75–85%. DONTEST IAC and SPE OASIS HLB cartridges were not suitable for the current study, because no recovery was observed by the former of 15Ac-DON or by the latter of FUS-X. QuEChERS and SLE show high ion suppression, e.g., 34% with DON-3Glc and 46% with 15Ac-DON, respectively. Consequently, the final procedure included clean-up with Mycosep 225 columns. Compared to other authors [15,39] the showed method allows for determination wider range of B-trichotecenes e.g., 15Ac-DON, NIV or FUS-X. Moreover, application for clean-up of sample Mycosep 225 is not as expensive as selective clean-up with IAC, which is frequently used for determination of DON and its metabolites in feeds [17,25,26].

### 2.4. Method Validation

The method was successfully validated for all tested mycotoxins in feedstuffs (Table 1). Good specificity of the methods was confirmed by analysis of 20 pseudo-blank samples. No interference peaks (S/N > 3) were detected in the retention time (±2.5%) for targeted analytes. The determined LOD and LOQ for all analytes were in the 1.78–15.0 and 5.87–49.5 µg/kg ranges, respectively. These low LOD and LOQ for DON and its modified mycotoxins were comparable with those disclosed in other publications [23,44], or even lower [45]. The calibration curves were linear over the calibration range for all compounds, resulting in R^2^ values between 0.998 and 0.999. The REC was determined for each toxin based on a sample fortified at three VL (0.5 × VL, 1.0 × VL, and 1.5 × VL) and was above 90%, which fulfilled established criteria [46]. All values for repeatability and within-laboratory reproducibility were in the range of 4–24%, showing good precision for all toxins. Moreover, RSDr was between 4% and 22%, indicating that this method could be adopted for a wide range of feedstuffs. The expanded measurement uncertainty U (%) was satisfactory at below 35% for all tested toxins, showing acceptable method performance. Due to the large variability and complexity of the analysed samples, significant ME was expected [47]. In our study, ME values ranged from 61% to 120%. To compensate for this enhanced or suppressed signal, matrix-matched calibration curves were used as well as IS for DON and DON-3Glc. The MIX IS was added after extraction only to compensate for possible ME, to limit use of the expensive IS. This result shows that the developed procedure can be applied as a confirmatory method for determination of DON, its metabolites and others type B trichothecenes in feedstuffs.

### 2.5. Method Trueness, PT

Method trueness was evaluated by analysing three RMs (Table 2) and comparing them with the reference values. For each matrix, DON concentrations were in the uncertainty range of each sample. It is worth noting, that RMs were naturally contaminated samples, so other mycotoxins were also found. For example in the RM M15362D maize sample (Chiron) DON-3Glc, 3Ac-DON, 15Ac-DON and NIV were quantified at levels of 431, 19.0, 202, 203 µg/kg, respectively. Moreover, the molar ratios between DON-3Glc and 15-Ac to DON were high (43% and 20%, respectively). These results also indicate that if we analyse RM contaminated in real circumstances it is highly probably that metabolites of DON can be found and these data could be used for evaluation of method trueness. In the case of a PT organised by the European Union Reference Laboratory mycotoxins and plant toxins, *z*-score results obtained for samples (wheat and maize) were in a tolerable range −2 ≤ z ≤ 2 (−0.48 and 1.43). Thus, the developed method can produce accurate results in accordance with the reference values and could be applied in future to real contaminated feedstuff samples. It is worth to mention that in this PT acetylated forms of DON and DON-3Glc were covered only by less than half and one third of the laboratories, respectively. Also, false positive and negative results were reported related to 15Ac-DON, what indicate that determination of isomeric form is a challenge to analytical researchers.

### 2.6. Application to Real Contaminated Feedstuffs

**Figure 5:**
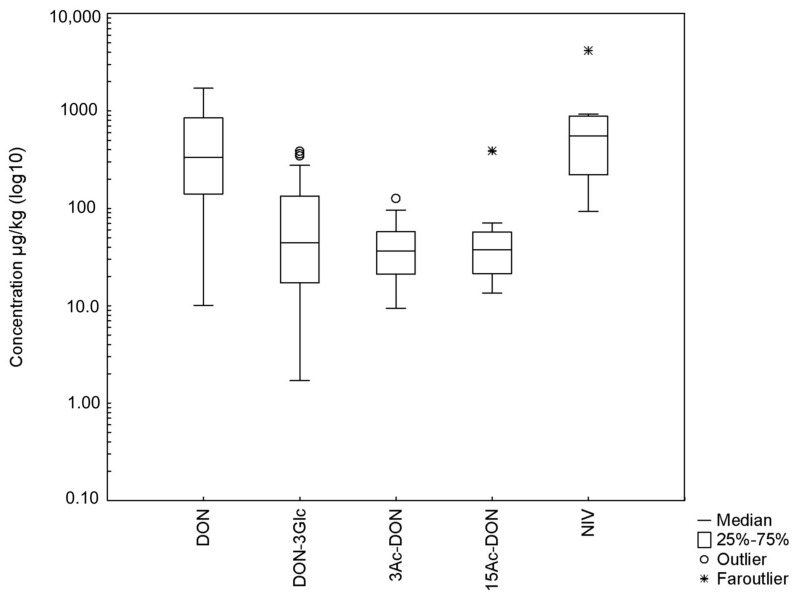
The mycotoxins concentrations in contaminated feedstuffs (*n* = 99).

The validated method was applied to the analysis of 99 feedstuff samples (Figure 5). DON and DON-3Glc were detected in 85% and 86% of surveyed samples and the mean concentrations were 511 µg/kg and 94.0 µg/kg, respectively (concentration ranging for DON between 10.1 and 1709 µg/kg and for DON-3Glc 1.70 and 385 µg/kg). Moreover, the ratio of the DON-3Glc concentration to that of DON ranged from 3% to 59% with a mean of 19%. The ratios of DON-3Glc/DON obtained in our study coincide with previously reported investigation [19,33]. The incidences of other toxins (3Ac-DON, 15Ac-DON, and NIV) were 35%, 26% and 23%, respectively.

## 3. Conclusions

A UHPLC-MS/MS method for the determination of DON, its metabolites and other type B trichothecenes in feedstuffs was successfully developed and validated. The main novelty of this method is that full separation of all compounds was achieved, including the isomeric forms 3Ac-DON and 15Ac-DON and that a DON-3Glc IS was used as the internal standard for quantification of DON-3Glc. In case of the IACs testing for they cross-reactivity features for DON modified forms none of them bound all derivatives and other toxins. The use of the commercially available Mycosep 225 columns allowed for quick and easy sample preparation. The results of RM analysis and the PT confirm the trueness of the method. Application of the validated method on feedstuffs revealed occurrence of DON and DON-3Glc in over 80% of positive samples. The developed method can be a tool for accurate qualification and quantification of mycotoxins and could be adopted as a confirmatory method for determination of DON and its modified mycotoxins NIV and FUS-X in a wide range of feedstuffs. 

## 4. Materials and Methods 

### 4.1. Chemicals and Standards

Six brands of IAC were compared for their cross-reactivity features: DONTest WB from Vicam, (Milford, MA, USA), DZT MS-PREP and DON PREP from R- Biopharm Rhone Ltd. (Glasgow, UK), B-TeZ IAC Deoxynivalenol from BioTeZ Berlin Buch GmbH (Berlin, Germany), DONStar from Romer Labs Diagnostic GmbH (Tulln, Austria) and aokinImmunoClean DON (DONaok) from Aokin AG (Berlin, Germany). DON PREP, B-TeZ IAC Deoxynivalenol and DONStar—were kindly provided free of charge by suppliers for testing purposes. Mycosep 225 Trich columns were purchased from Romer Labs Diagnostic GmbH. Oasis HLB cartridges were obtained from Waters (Milford, MA, USA). Acetonitrile (analytical and LC-MS grade; ACN), methanol (LC-MS grade; MeOH), acetic acid and C18 bulk sorbent were sourced from J.T. Baker of Avantor Performance Materials (Deventer, The Netherlands). Magnesium sulphate (MgSO_4_) was from Chempur (Piekary Śląskie, Poland) and water was prepared using a Milli-Q apparatus (MerckMillipore, Burlington, MA, USA) to attain purity of 18.2 MΩ. Mycotoxin standards of DON, U-[13C15] DON (DON IS), 3Ac-DON, 15Ac-DON, NIV and FUS-X were obtained from Sigma Aldrich (St. Louis, MO, USA). DON-3Glc and U-[13C21] DON-3G (DON-3Glc IS) were purchased from Romer Labs. The primary standard stock solutions were prepared in ACN. All standards were stored according to their manufacturer’s recommendations. The chloride and pottassioum chloride used to make phosphate buffered saline (PBS) were sourced from POCh (Gliwice, Poland) and the sodium hydrophosphate dehydrate from Chempur. PBS was made as follows: 8 g of sodium chloride, 3.6 g of sodium hydrophosphate dihydrate and 0.2 g of potassium chloride were dissolved in 1L of deionized water.

### 4.2. Mixed Working Solution

A mixed working solution (MIX6) was prepared in ACN from the individual stock of six mycotoxins at a concentration of 9 μg/mL for DON and NIV and 1 μg/mL for 3Ac-DON, 15Ac-DON, DON-3Glc and FUS-X. The internal standards solution (MIX IS) was mixed in ACN to achieve concentrations of 1 μg/mL and 0.5 μg/mL for DON IS and DON-3Glc IS, respectively. All working standard solutions were stored at 2–8 °C.

### 4.3. Samples and Reference Materials

**Figure 6:**
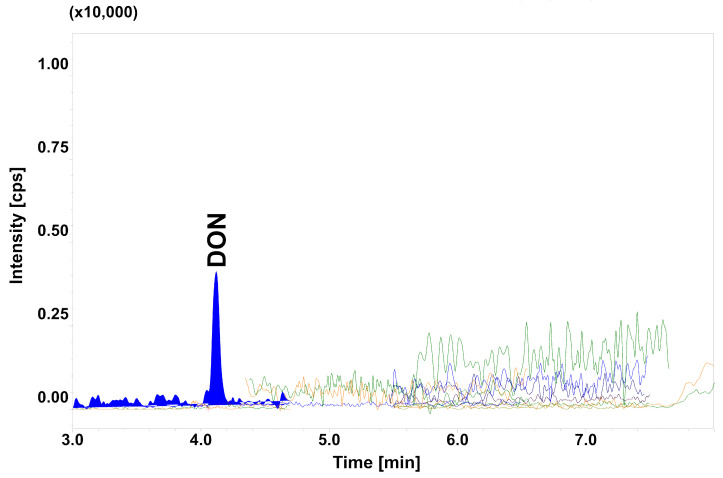
Chromatogram of pseudo-blank sample with DON concentration of 50 ± 13 μg/kg.

Poultry and swine feedstuff samples (total *n* = 99) were collected in 2017 and 2018 by Veterinary Inspectorate officers working with feed manufacturers, in accordance to Commission Regulation (EC) NO. 2009/152 [48]. Delivered samples were milled using a ZM 200 ultra-centrifugal high-speed instrument with 1 mm sieve (Retsch GmbH, Haan, Germany;) and stored in a dark place at room temperature until analysis. A validation study was conducted using low contamination feed samples (pseudo-blanks) [49] with DON concentration of 50 ± 13 μg/kg (Figure 6). 

For confirmation of the method trueness, three reference materials (RMs) were tested: maize (TET030RM; Fapas, Fera Science, York, UK), maize (12199.15-G; Chiron AS, Trondheim, Norway) and wheat (TET007RM; Fapas) (Table 2). Moreover, the method was verified in the “Deoxynivalenol and related compounds in food and feed matrices”; EURLPT-MP01 proficiency test on DON, 3Ac-DON, 15Ac-DON and DON-3Glc in wheat and maize carried out by the European Union Reference Laboratory mycotoxins and plant toxins.

### 4.4. IAC Testing 

The cross-reactivity of six selected IAC was evaluated by loading 4 mL of water or PBS (according to the manufacturer’s recommendation) spiked with the MIX 6 mixed mycotoxin solution (at the level at which the analytes concerned could be extracted from samples). Next, the IAC was flushed with 4 mL of water and analytes were eluted with two portions of 1.5 mL of MeOH. The solvent was evaporated to dryness under a nitrogen stream at 40 °C and the dry residue was dissolved in 200 µL of 0.2% acetic acid and transferred to an autosampler vial. Each IAC was tested in triplicate. For comparison of their suitability for detection of the relevant mycotoxins, recovery was calculated as the ratio of the measured concentration of toxins to neat solvent.

### 4.5. Compared Strategies for Sample Preparation and Clean-Up

In addition to IACs, several other techniques of sample preparation have been tested: SLE, QuEChERS, SPE with OASIS HLB cartridges and Mycosep 225 Trich column (contain a mixture of adsorbent materials). The protocol for sample preparation for SLE and QuEChERS was based on our previously described methods [41,42].

The OASIS HLB column was tested according to the manufacturer’s protocol. Briefly, 1 g of sample was weighed into a 50 mL plastic tube and extracted for 30 min with 8 mL of H_2_O. Next, the sample was centrifuged and 2 mL of extract was transferred into OASIS HLB cartridges previously conditioned with 3 mL of MeOH and 3 mL of H_2_O. Subsequently, the column was washed with 5 mL of 5% MeOH and eluted with 3 mL of MeOH. All sample were collected and evaporated to dryness under a gentle nitrogen stream at 40 °C. The residue was dissolved in 200 µL of 0.2% CH_3_COOH and transferred to an autosampler vial. 

For IAC DONTEST 1 g of sample was weighed into a 50 mL plastic tube and extracted for 30 min with 8 mL of H_2_O. Next, the sample was centrifuged and 2 mL of extract was passed onto DONTEST. Subsequently, the column was washed with 10 mL water and finally eluted with 2 mL methanol and collected to glass tube. After evaporation (N_2_, 40 °C) sample was dissolved in 200 µL of 0.2% CH_3_COOH and transferred to an autosampler vial.

For the sample preparation using for clean-up Mycosep 225 Trich column (based on the manufacturer’s recommendation with slight modification) 1 g of previously milled sample was weighed into a 50 mL centrifuge tube. Next, 8 mL of ACN:H_2_O (84:16; *v*/*v*) mixture was added and was extracted in a rotary shaker for 30 min, followed by centrifugation for 15 min at 4000 rpm. Subsequently, 6 mL of supernatant was transferred to a glass tube and pushed through a Mycosep 225 Trich column. Purified extract (2 mL) was collected, and the sample was evaporated to dryness in a gentle nitrogen stream at 40 °C. The dry residue was reconstituted in 200 µL of 0.2% CH3COOH and transferred to an autosampler vial. For final optimized sample preparation additionally, 10 µL of MIX IS was added before sample evaporation. Extraction recovery (ER) and matrix effect (ME) were calculated to find an appropriate method for sample preparation. In this case, ER was calculated as the ratio of the area of the analyte(s) recorded for the sample spiked with the target compound(s) before extraction to the area for the spiked sample after extraction.

### 4.6. LC-MS/MS Analysis

The analysis was performed with a Nexera X2 system with an LCMS-8050 triple-quadrupole mass spectrometer (Shimadzu, Kioto, Japan). LabSolutions software (version 5.60 SP2, Shimadzu, Kioto, Japan) was used for data acquisition and processing. Chromatographic separation was tested using four chromatographic columns with the different stationary and mobile phase composition (Table S2). Column and autosampler temperatures were set at 45 °C and 4 °C, respectively. The final optimised mobile phase A consisted of 0.2% CH3COOH in water/ACN (95:5; *v*/*v*) (eluent A) and ACN/0.2% CH3COOH in water (95:5; *v*/*v*) (eluent B). A gradient elution was used as follows: 0–2 min 15% B, 2.1–6 min 18% B, 6.1–9 min isocratic step at 100% B, and 9.1–12 min 0% B. The total run time was 12 min at a flow rate of 0.3 mL/min and the injection volume was 5 µL. 

The mass spectrometry detection was carried out using multiple reaction monitoring (MRM) with positive and negative electrospray ionization (ESI+/−) (Table S2). The following ion-source settings were used: nebulising gas flow: 2 L/ min, heating gas flow: 10 L/min, drying gas flow: 10 L/min, interface temperature: 300 °C, desolvation line temperature: 250 °C, heat block temperature: 400 °C, and Q1 and Q3 resolution: Unit. 

The identification of the analyte was performed according to the SANTE/12089 /2016 Guidance document on identification of mycotoxins in food and feed [50]. Four identification criteria were used: comparison of peak retention time in test samples with retention times of calibration standards; the retention time of the internal standard being within the tolerance range ±0.05 min relative to the appropriate standard; selection of at least two characteristic fragmentary ions and calculation of their ion ratio (within ±30% (relative) of average of calibration standards from the same sequence); and the peaks having an S/N ratio of at least 3. 

### 4.7. Method Validation

The method was validated for feedstuffs and the following parameters were verified and calculated: specificity, limit of detection (LOD) and quantification (LOQ), linearity, apparent recovery (REC, %), precision as repeatability (RSD, CV%) and within-laboratory reproducibility (RSDr, CV%), trueness and matrix effect (ME, %) [46,50]. All validation parameters were calculated using the relative peak area with respect to DON IS and DON-3Glc IS. The specificity was checked by analysing 20 different pseudo-blank feed samples to evaluate possible interferences. LOD and LOQ were also calculated based on Guidance Document on the Estimation of LOD and LOQ for Measurements in the Field of Contaminants in Feed and Food using paired observation approach [27]. The three validation levels (VL) 0.5 × VL, 1.0 × VL, and 1.5 × VL used for DON and NIV were: 450, 900, and 1350 µg/kg, and for DON-3Glc, 3Ac-DON, 15Ac-DON and FUS-X they were: 50, 100, and 150 µg/kg. The decisions on fortification levels for each analyte were made on basis of the lowest guidance value EU in feedstuffs (only for DON, 900 µg/kg) [8]. For other toxins—based on concentration data in feedstuffs reported by others authors [13,18,35] and LC-MS/MS detection. The linearity was determined using a matrix-matched calibration plot. The concentration ranges of the five-point calibration curves were 90–1800 μg/kg for DON and NIV and 10–200 μg/kg for DON-3Glc 3Ac-DON, 15Ac-DON and 50–200 μg/kg for FUS-X. The REC (apparent recovery) was calculated by quantifying the mycotoxins using matrix-matched calibration curves at the three VL. For the repeatability study, one kind of pseudo-blank feedstuff was spiked at three levels in six repetitions. The within-laboratory reproducibility was assessed by analysis of six different feedstuffs for animals (wheat, maize, feedstuffs for pigs, poultry, fish and mixed cereals) at the three VL on different days by two operators. Trueness was evaluated by analysing three RM in duplicate (Table 2).

To evaluate the ME, six different pseudo-blank samples were extracted with the proposed procedure and spiked after extraction with pure standards of all tested mycotoxins at the same level as in the within-laboratory reproducibility study. The responses of the mycotoxins were compared to a neat standard solution. With such a calculation, the ratio of 1:1 (100%) would mean no matrix effects, ion enhancement would result in a ratio above 100%, and ion suppression in a ratio below 100%. To compensate for possible losses caused by ME, DON IS was used for quantification of DON, NIV, 3-AcDON, 15Ac-DON, FUS-X and DON-3Glc IS for DON-3Glc. Additionally, the expanded measurement uncertainty (U) was calculated with MUkit software (Envical SYKE, Helsinki, Finland) for the 1.0 × VL spiking level using the Nordtest approach [51].

## Figures and Tables

**Table 1:** Validation parameters for the analysed mycotoxins in feedstuffs.

	REC (%)			ME (%)			Calibration Range(µg/kg)	R^2^		Precision (CV, %)					LOD (µg/kg)	LOQ (µg/kg)	U (%)
									RSD			RSD_r_					
	0.5 × VL	1.0 × VL	1.5 × VL	0.5 × VL	1.0 × VL	1.5 × VL			0.5 × VL	1.0 × VL	1.5 × VL	0.5 × VL	1.0 × VL	1.5 × VL			
DON	104 ± 4	93 ± 6	99 ± 6	82 ± 12	101 ± 17%	87 ± 8	90–1800	0.999	9	9	11	4	7	6	10.1	33.3	26.0
DON-3Glc	93 ± 10	92 ± 12	96 ± 10	78 ± 12	61 ± 15%	74 ± 14	10–200	0.999	20	8	13	10	15	10	1.78	5.87	12.0
3Ac-DON	105 ± 12	94 ± 6	104 ± 10	109 ± 7	119 ± 20%	113 ± 7	10–200	0.999	9	9	9	12	7	10	2.43	8.02	26.0
15Ac-DON	97 ± 12	92 ± 16	90 ± 18	115 ± 15	120 ± 16%	111 ± 12	10–200	0.999	4	9	13	22	19	21	5.65	18.6	25.0
NIV	106 ± 14	97 ± 15	94 ± 15	96 ± 6	85 ± 7%	89 ± 6	90–1800	0.999	16	13	24	13	19	17	3.30	10.9	25.0
FUS-X	101 ± 12	93 ± 12	96 ± 11	93 ± 6	104 ± 15%	88 ± 8	50–200	0.998	6	9	10	8	16	14	15.0	49.5	35.0

**Table 2:** Trueness of results obtained by the developed method; mycotoxins concentration determined in proficiency testing materials (EURL) and RMs.

Reference Sample	Matrix	Analyte	Reference Concentration (µg/kg)	Concentration Uncertainty (µg/kg)	Measured Concentration (µg/kg) ^a^	Calculated *z*-Score
Proficiency Test EURLPT-MP01	wheat	DON	570	±14.8	502	−0.48
		DON-3Glc	215	±22.2	217	−0.04
		3Ac-DON	35.2	±2.40	32.0	−0.37
	maize	DON	751	±20.4	729	−0.12
		DON-3Glc	35.0	±1.48	47.5	1.43
		3Ac-DON	92.8	±3.71	90.2	−0.11
		15Ac-DON	152	±11.5	159	0.18
RM M15362D (Chiron)	maize	DON	1077	±73.0	993	---
		DON-3Glc	---	---	431	---
		3Ac-DON	---	---	19.0	---
		15Ac-DON	---	---	202	---
		NIV	---	---	203	---
RM TET007RM (Fapas)	wheat	DON	1810	±311	1956	---
		DON-3Glc	---	---	125	---
RM TET030RM (Fapas)	maize	DON	1208	±117	1077	---
		DON-3Glc	---	---	190	---
		15Ac-DON	---	---	121	---

^a^ measured in duplicate

## Supplementary Materials

The following are available online at https://www.mdpi.com/2072-6651/12/6/362/s1, Table S1: LC-MS/MS parameters for detection of mixed mycotoxins by the mass spectrometer; Table S2: LC column used during the chromatographic set up.
