# Supplementary material for: Simultaneous Determination of Deoxynivalenol, Its Modified Forms, Nivalenol and Fusarenone-X in Feedstuffs by the Liquid Chromatography–Tandem Mass Spectrometry Method
Source: Toxins (Basel). 2020 Jun 1;12(6):362. doi: 10.3390/toxins12060362 (PMC7354445; doi:10.3390/toxins12060362)
Supplement: Supplementary file 1 [file toxins-12-00362-s001.pdf]

Supplementary Materials: Determination of Deoxynivalenol, Its Modified Forms, Nivalenol and Fusarenon-X in Feedstuffs by the Liquid Chromatography–Tandem Mass Spectrometry Method

Łukasz Panasiuk , Piotr Jedziniak, Katarzyna Pietruszka and Andrzej Posyniak

Table S1. LC-MS/MS parameters for detection of mixed mycotoxins by the mass spectrometer.

Analytes	Precursor ion form	Precursor ion *m/z*	Product ion (m/z)	Collision energy (eV)	Dwell time (msec)	Retention time (min)
**NIV**	[CH3COO⁻]	371.0	281.2*	17	25	3.4
			311.1	12		
			59.2	24		
**DON-3Glc**	[CH3COO⁻]	517.0	427.0*	20	25	3.9
			215.2	33		
			457.0	15		
**U-[$^{13}C_{21}$] DON-3Glc**	[CH3COO⁻]	538.0	447.2	24	25	3.9
			478.2	17		
**DON**	[CH3COO⁻]	355.1	295.2*	11	25	4.1
			265.2	16		
			59.2	30		
**U-[$^{13}C_{15}$] DON**	[CH3COO⁻]	370.0	310.2	11	25	4.1
			279.1	16		
**FUS-X**	[H⁺]	355.0	337.1*	-10	50	4.9
			229.1	-18		
			175.2	-22		
**3-AcDON**	[H⁺]	339.0	231.2*	-14	30	7.1
			279.1	-15		
**15-AcDON**	[H⁺]	339.0	321.2*	-10	30	6.8
			261.2	-12		

*- ion used for quantitation.

Table S2. LC column used during the chromatographic set up.

Columns	Stationary Phase	Particle Size (µm)	Pore Size (Å)	Length (mm)	Internal Diameter (mm)
Phenomenex Luna Omega	Polar C18	1.6	100	100	2.1
Phenomenex Kinetex	C18	2.6	100	100	2.1
Phenomenex Kinetex	Biphenyl	1.7	100	100	2.1
Phenomenex Luna	HILIC	5	200	100	2
